# Sweet Syndrome Associated With Crohn’s Disease Developed After a Combo Therapy With Infliximab and Azathioprine: A Case Report

**DOI:** 10.7759/cureus.20749

**Published:** 2021-12-27

**Authors:** Christos Sotiropoulos, Eftichia Sakka, Georgios J Theocharis, Konstantinos C Thomopoulos

**Affiliations:** 1 Gastroenterology, University General Hospital of Patra, Patra, GRC; 2 Internal Medicine, General Hospital of Patra "St Andrew", Patra, GRC

**Keywords:** skin lesions, extra-intestinal manifestations, crohn’s disease, inflammatory bowel disease, sweet syndrome

## Abstract

Sweet syndrome, also known as Acute Febrile Neutrophilic Dermatosis, is a rare inflammatory condition. The exact pathogenesis of Sweet syndrome is unclear, however, autoimmune and inflammatory conditions including inflammatory bowel disease have been linked as underlying etiologies. Since its description, in 1964, there have been published less than fifty reports of Crohn’s-associated Sweet syndrome.

We report a 43-year-old male patient with a medical history of Crohn’s disease who subsequently developed Sweet syndrome. Two years after the diagnosis of Crohn’s disease the patient was administered a combo therapy with Infliximab and Azathioprine followed by deep remission. A few months later the patient manifested with skin lesions with histopathological findings suggestive of Sweet syndrome.

Sweet syndrome, although rare, may occur as an extra-intestinal manifestation of Crohn’s disease. This report illustrates the need for a thorough investigation of patients with Crohn’s disease presenting with skin lesions. We hope it will add to the current literature and help understand this rare phenomenon in order to achieve a proper diagnosis.

## Introduction

Ιnflammatory bowel disease (IBD) includes Crohn’s disease (CD) and ulcerative colitis (UC) [1]. CD typically manifests with inflammatory skip lesions all over the gastrointestinal tract, with the small intestine being the first target [1]. CD can also present with extraintestinal manifestations such as dermatologic, ocular, cutaneous, and joint features [1]. Pyoderma gangrenosum and erythema nodosum are frequent cutaneous manifestations, while Sweet syndrome constitutes a rare clinical feature of CD [1].

Sweet syndrome is an acute febrile neutrophilic dermatosis that was first described by Robert Douglas Sweet in 1964 [2, 3]. It is a non-infectious disorder that is developed by neutrophilic infiltration of the skin [2, 3]. Sweet syndrome is characterized by the onset of well-defined tender plaques or nodules, fever, ocular inflammation, arthralgias, headaches, and oral or genital lesions [2, 3].

Most commonly, Sweet syndrome is idiopathic but in rare cases, it can be secondary to an underlying disorder such as malignancy or inflammatory and autoimmune disease [2]. According to the etiology it can be categorised as idiopathic, paraneoplastic, drug-induced, para-inflammatory, or pregnancy-associated [4]. Autoimmune and inflammatory disorders including inflammatory bowel disease (ulcerative colitis and Crohn’s disease) have been directly associated as underlying etiologies [4]. However, less than 50 cases of Crohn’s-associated Sweet syndrome have been reported in the published literature so far [4].

In addition, Sweet syndrome can be drug-induced [2]. Granulocyte-colony stimulating factor (G-CSF), antibiotics (norfloxacin, trimethoprim-sulfamethoxazole), antihypertensives (furosemide), non-steroidal anti-inflammatory drugs (NSAIDs), immunosuppressives (azathioprine), anti-cancer drugs, and antipsychotics are the most commonly related drug categories [2].

This report presents a rare case of Sweet syndrome in a patient with CD developed after treatment with infliximab (IFX) and azathioprine (AZA).

## Case presentation

We present a 43-year-old male patient with a medical history of Crohn’s disease (diagnosed in 2009) who developed various extra-intestinal manifestations. At the age of 32, the patient was referred to our clinic due to a history of severe bloody diarrhoea (five to six watery stools per day), hemodynamically stable, with colic abdominal pain, arthralgia, and unintentional weight loss. He underwent colonoscopy which revealed a discontinuous distribution of longitudinal ulcers and cobblestone appearance of the rectum and sigmoid colon, pseudopolyps, and colonic stenosis approximately 30 cm from the anal verge, with impossible further advancement of the endoscope. Biopsies from the colon were suggestive of IBD, with Crohn’s disease being the most probable diagnosis. At the presentation, the patient's scores were the following: Crohn's Disease Activity Index: 310 points, Harvey-Bradshaw Index: 12 points (moderate disease), Simple endoscopic score for Crohn's disease: 16 points (severe endoscopic activity).

Two years later (in 2011), after an initial treatment with methylprednisolone 32 mg once a day and mesalazine 1 g three times a day, the patient was hospitalized for encircled intestinal perforation, confirmed with a Computed Tomography scan, which was treated conservatively with nothing by mouth, bowel rest and intravenous antibiotics. After a few days of uncomplicated hospitalization the patient was discharged with a low dose of methylprednisolone (8 mg daily).

**Figure 1:**
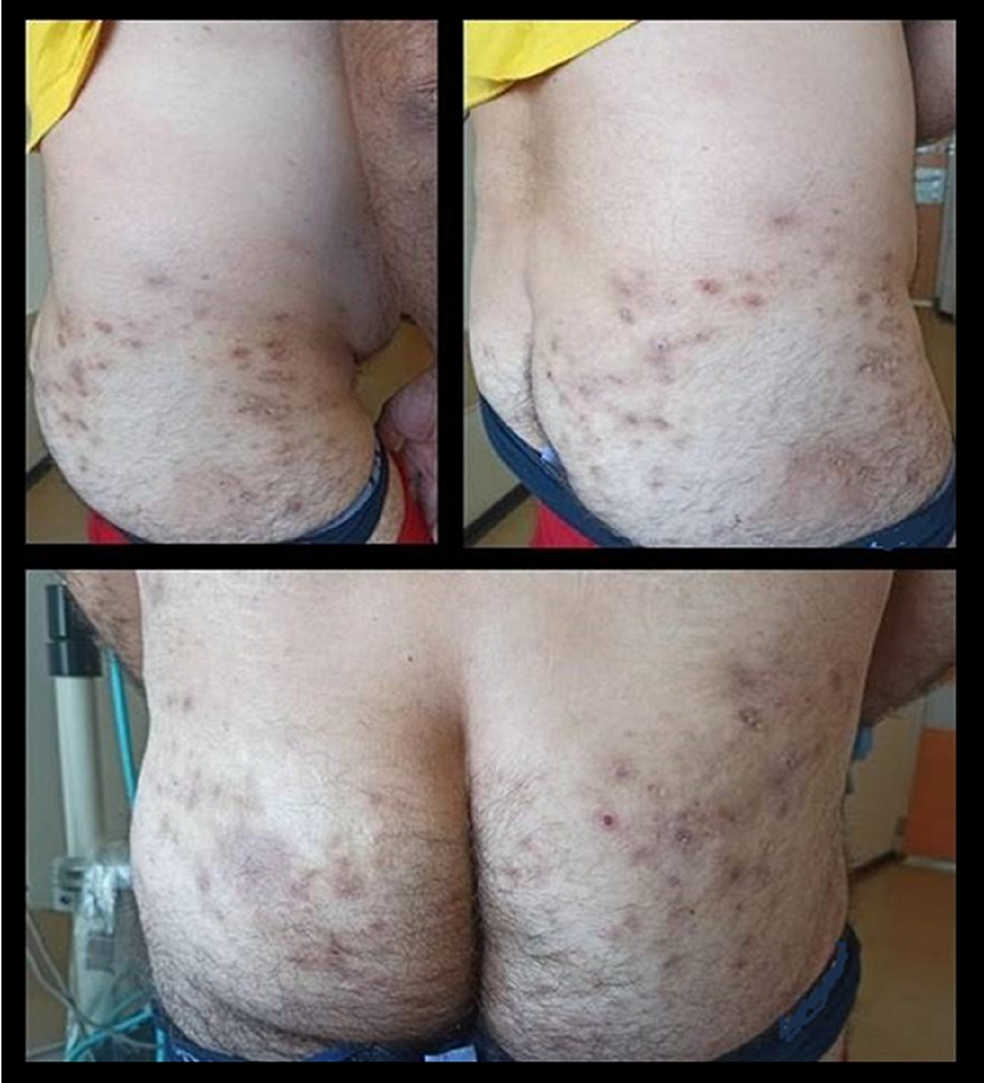
Defined tender plaques and nodules in the pelvis, hip, gluteus and lower limbs.

One year later (in 2012), while being on a long-time remission without therapy, the disease exacerbated and the patient was administered a combo therapy with IFX and AZA. Three months later, he manifested arthralgias, fever (>38^o^C), and well-defined tender plaques and nodules in the pelvis, hip, gluteus, and lower limbs (Figure 1).

**Figure 2:**
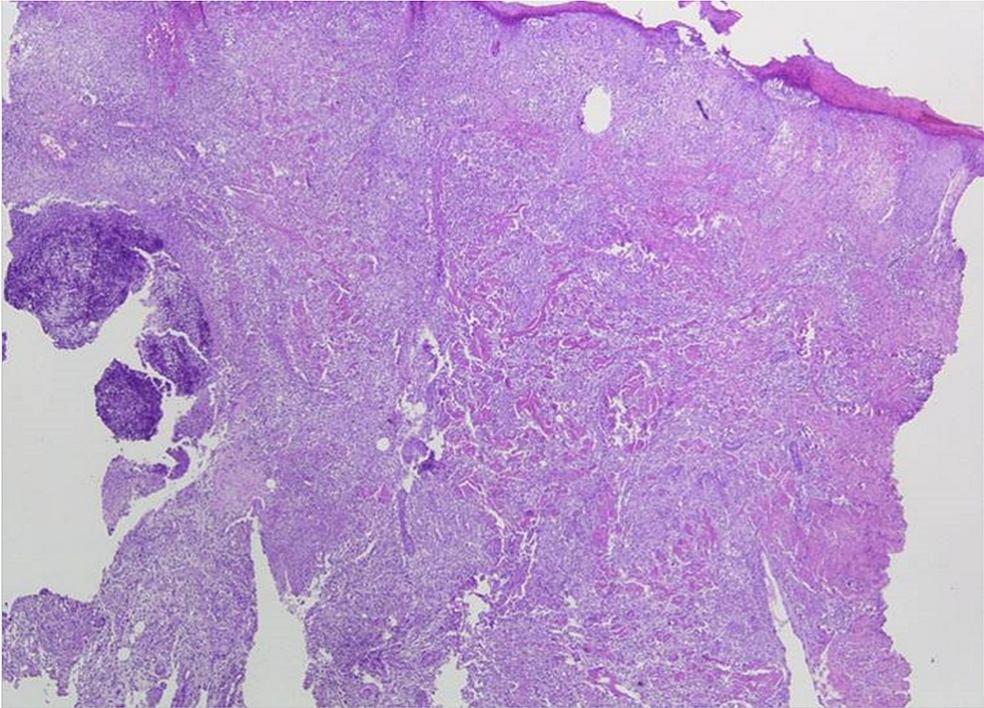
Histopathological evaluation of skin biopsies revealing diffuse neutrophilic infiltrate in the reticular dermis, perivascular lymphocyte infiltrations and pseudoepithelial hyperplasia of the skin.

Laboratory tests revealed slight elevation of white blood cells (WBC: 11,59 K/μl, neutrophils>80%) and C-reactive protein (CRP: 4,13 U/l), abnormal erythrocyte sedimentation rate (>20), and immunology investigations (rheumatoid factor [RF], complement component 3 [C3], complement component 4 [C4], antinuclear antibodies [ANA], anti-neutrophil cytoplasmic antibodies [ANCA], anti-mitochondrial antibodies [AMA], anti-smooth muscle antibodies [ASMA], anti-saccharomyces cerevisiae antibodies [ASCA]). A dermatologic evaluation was ordered and skin biopsies were performed. The histopathological findings revealed a diffuse neutrophilic infiltrate in the reticular dermis, perivascular lymphocyte infiltrations, and pseudoepithelial hyperplasia of the skin (Figure 2) suggestive of Sweet syndrome.

Until now, the previous medication was stopped and the patient was treated for the skin lesions with several drugs and therapeutic agents (corticosteroids, immunosuppressants, immunomodulators) unsuccessfully. Today, he is in deep remission, treated with an interleukin inhibitor (ustekinumab), with frequent exacerbations and relapses of skin lesions unresponsive to treatment.

## Discussion

Sweet syndrome appears more commonly in women than men (4:1) with the highest frequency between 30 and 60 years of age [1, 5]. The typical clinical and laboratory findings include fever, sudden-onset of painful papules, plaques, and skin nodules affecting the face, neck, or upper limbs, leukocytosis, and diffuse infiltration of neutrophils in the upper dermis [1, 3, 4].

The diagnosis is confirmed when both major criteria and two out of four minor criteria are fulfilled [1, 4, 5]. Major criteria consist of (a) sudden onset of painful plaques or skin nodules and (b) histopathologic evidence of neutrophilic infiltrate without leukocytoclastic vasculitis [1, 4, 5]. Minor criteria consist of (a) pyrexia >38^o^C, (b) association with a haematologic or visceral malignancy, pregnancy, or inflammatory disease, preceded by a gastrointestinal or an upper respiratory infection, or vaccination, (c) excellent response to corticosteroid or potassium iodide treatment and (d) abnormal laboratory values, erythrocyte sedimentation rate >20 mm/h, positive C-reactive protein, leukocytes >8000, neutrophils >70% (three out of four needed) [1, 2, 5].

The classic histopathologic pattern of Sweet syndrome consists of a diffuse neutrophilic infiltrate in the reticular dermis [2]. Eosinophils and lymphocytes may be present, but neutrophils predominate [1]. Although subtle vasculitic changes may be present, true vasculitic changes usually are absent [2].

Corticosteroids, potassium iodide, and colchicine are first-line treatments, while second-line treatments include indomethacin, dapsone clofazimine, doxycycline, and cyclosporine [1]. Sweet syndrome can be a rare extraintestinal manifestation of CD [1]. There are only a few case reports of Sweet syndrome related to CD and its occurrence seems to correlate with gastrointestinal activity [1]. Therefore, while evaluating patients manifesting with skin lesions and associated gastrointestinal complaints, clinicians have to consider IBD as a leading pathology in differential diagnosis [5].

The reported patient had uncontrolled CD for two years prior to starting IFX infusions and AZA. A few months later, he manifested dermatologic lesions with biopsies suggestive of Sweet syndrome, highlighting a possible drug-induced aetiology. Today, while being on deep remission treated with an interleukin inhibitor, the skin lesions are unresponsive to corticosteroids and other agents.

To the best of our knowledge, less than 50 cases of Sweet syndrome associated with IBD have been reported, mostly with CD. We report a case to support a novel association between Crohn’s disease and Sweet syndrome in the context of a combo therapy with IFX and AZA, as well as to alert physicians to the importance of this rare entity.

## Conclusions

Although Sweet syndrome is rare, relations with medications, inflammatory diseases and malignancy have been established and expanded on. It is important to consider Sweet syndrome a possible extraintestinal manifestation of Crohn’s disease in patients already diagnosed or suspected for IBD, but also a possible side effect of the drugs usually used in IBD patients. This report highlights the need for clinicians to place IBD high in the differential diagnosis in patients presenting with concurrent cutaneous and gastrointestinal complaints.
